# Next-generation photo-Fenton treatment using MIL-100(Fe) synthesized through a green route for sustainable remediation of pharmaceutical wastewater

**DOI:** 10.1038/s41598-026-38975-w

**Published:** 2026-03-01

**Authors:** Ahmed S. Abou-Elyazed, Eman E. Genena, Ibrahim E. T. El-Sayed, Hamed M. Abdel-Bary, Maha A. Tony

**Affiliations:** 1 Chemistry Department, Faculty of Science, Menoufia University, Shebin El- Kom, 32512 Egypt; 2 Advanced Materials/Solar Energy and Environmental Sustainability (AMSEES) Laboratory, Basic Engineering Science Department, Faculty of Engineering, Menoufia University, Shebin El-Kom, 32512 Egypt; 3 Planning & Construction of Smart Cities Program, Faculty of Engineering, Menoufia National University, 32651 Menoufia, Egypt

**Keywords:** Photo-Fenton-process, Ecofriendly MIL-100(Fe), Paracetamol wastewater, Oxidation mechanism, Chemistry, Environmental sciences, Materials science

## Abstract

The photocatalytic Fenton oxidation reaction of paracetamol (PCT), as a widely used pharmaceutical and persistent water contaminant, was investigated using RTG-MIL-100(Fe) framework, prepared via a KI-assisted, solvent free, room temperature synthesis, in the presence of H₂O₂ under UV irradiation. The mild conditions synthesis of RTG-MIL-100(Fe) is investigated, and the material is comprehensively assessed through characterized using X-ray Diffraction (XRD), Scanning Electron Microscopy (SEM), Brunauer–Emmett–Teller (BET), and X-ray Photoelectron Spectroscopy (XPS). The catalytic system is introduced as a source of photo-Fenton-like reaction and demonstrated a dual mechanistic pathway involving both semiconductor-like photoexcitation and open metal site (OMS) activation. The operating Fenton parameters are optimized and the experimental research revealed that the reaction is working at the room temperature using 400 ppm H₂O₂, 20 mg/L of RTG-MIL-100(Fe)at natural pH of wastewater (5.5) and the PCT reached almost complete removal 99.6%. The synergistic interaction of photogenerated charge carriers and OMS-driven Fenton-like processes resulted in efficient oxidative mineralization of PCT to CO₂ and H₂O. Furthermore, the reaction is endothermic in nature and following the first order reaction kinetics. These findings highlight the potential of KI-modified MOF-based photo-Fenton catalysts as sustainable and robust materials for the treatment of pharmaceutical wastewater.

## Introduction

Water scarcity and freshwater pollution continue to threaten global water security, largely due to the release of persistent organic contaminants into aquatic environments^1–5^. Among these emerging pollutants, pharmaceutical residues, particularly paracetamol (PCT), have gained significant attention owing to their extensive consumption, chemical stability, poor biodegradability, and frequent detection in wastewater and natural water bodies^6–13^. Because of its high solubility and low metabolic conversion in humans, unmetabolized PCT is continuously discharged into the environment, where it can induce reproductive toxicity, genotoxicity, organ damage, and broader ecological disturbances^10–13^. Consequently, the effective removal of PCT and related pharmaceuticals has become a critical priority for protecting water quality and public health^14–17^. Numerous studies have reported the rising environmental risks associated with pharmaceutical pollutants, particularly the ecotoxicological impact of paracetamol residues in aquatic ecosystems^18–20^. Given its toxicity and environmental persistence, eliminating PCT and its byproducts from water is critical for ensuring safe and clean drinking water^21–24^. Recent reports^21–25^ indicate a sharp rise in the environmental occurrence of paracetamol and other pharmaceutical residues, with concentrations frequently detected in surface waters, wastewater effluents, and even drinking water sources^26,27^. These emerging findings highlight the growing ecological and public-health risks associated with pharmaceutical pollution, underscoring the urgent need for efficient and sustainable treatment technologies. Advanced oxidation processes (AOPs) such as UV/H₂O₂, UV/O₃, TiO₂ photocatalysis, and Fenton-based systems have been emerged as substituting the conventional techniques^21^. Notably, recent advances highlight hybrid oxidation systems, activated carbon–based photocatalysts, and TiO₂-based photoanodes as powerful approaches for accelerating oxidative degradation pathways^20–22^.

Metal–organic frameworks (MOFs) have recently gained prominence as next-generation catalysts for pollutant degradation due to their tunable porosity, high surface area, and rich redox activity^28–35^. Among them, MIL-100(Fe) is particularly attractive for Fenton and photo-Fenton applications because of its accessible iron-oxo clusters, structural robustness, and low cost^36^. Despite these advantages, key challenges remain: most MIL-100(Fe) syntheses still depend on harsh or solvent-intensive conditions, and the influence of halide additives particularly iodide on MOF formation, defect generation, and catalytic activation is not well understood^37^. Although numerous studies have investigated MOFs for the removal of pharmaceuticals, several critical limitations remain unresolved. First, most reported MOF syntheses still rely on organic solvents, high temperatures and long reaction times, which limit scalability and environmental sustainability. Second, truly green, solvent-free, room-temperature routes are still rare, and their influence on structural, textural and catalytic properties is not fully clarified. Third, the specific role of halide additives such as KI in directing MOF nucleation, porosity and catalytic activation remain underexplored for MIL-100(Fe)^37^.

To address these gaps, this study investigates a KI-modified MIL-100(Fe) catalyst for enhanced photo-Fenton degradation of PCT. A novel combination of hydrothermal synthesis and room-temperature, solvent-free iodide incorporation was explored to evaluate the role of KI in tuning morphology, porosity, Fe³⁺/Fe²⁺ redox cycling, and reactive oxygen species generation. The resulting materials were systematically characterized and assessed for photocatalytic degradation under optimized conditions. This work provides new insights into halide-assisted modification of MIL-100(Fe) and demonstrates a sustainable strategy for developing efficient MOF-based catalysts for pharmaceutical wastewater treatment.

## Experimental

### Materials

1,3,5-Benzenetricarboxylic acid (H_3_BTC, 98%) and potassium iodide (KI, 99%) were purchased from Shanghai Macklin Biochemical Co. Ltd. Ferric nitrate nonahydrate (98%) was obtained from Tianjin Kemi Chemical Reagent Co. Ltd. and ethanol (99.7%, Sinopharm, China) are all used for synthesis. For pHpzc determination, sodium chloride (99.0%, Sigma-Aldrich) is used. All of chemicals are of analytical grade and used as received with no further purification or treatment. Moreover, for synthetics wastewater preparation, analytical grade paracetamol (N-acetyl-para-aminophenol (APAP)) that is supplied by Aladdin Shanghai Co., Ltd, used to prepare the aqueous stock solution. Also, H_2_SO_4_ (98%, Sinopharm, China), HCl (37%, Sinopharm, China) and NaOH (99%, LOBA CHEMIE, India) are used for solutions pH adjustment. For the photo-Fenton reaction experiment, hydrogen peroxide (H_2_O_2_, 30% w/v) was used to initiate the reaction.

### Catalyst preparation

#### KI-assisted post-synthetic modification of MIL-100(Fe)

MIL-100(Fe) was synthesized according to our previous work^33^ and consistent with other reported procedures for MIL-100(Fe) preparation^38^. In a typical synthesis, Fe(NO₃)₃·9 H₂O, H₃BTC, and deionized water were mixed in a 50 mL two-neck round-bottom flask equipped with a magnetic stir bar and a reflux condenser. The mixture was heated at 95 °C under stirring for 24 h. The reaction product was centrifuged and rinsed three times with hot water and ethanol at 70 °C for 1 h, then dried in an oven at 100 °C for 3 h and activated under vacuum at 150 °C for 24 h. After synthesis, the resulting solid was subjected to a post-synthetic modification process using potassium iodide (KI); the MIL-100(Fe) powder was dispersed in a series of aqueous KI solutions with different concentrations: 0.1 M, 0.01 M, and 0.001 M. Each dispersion was stirred continuously at room temperature for 24 h to promote interaction between KI and the MIL-100(Fe) structure. KI doping enhances Fe³⁺/Fe²⁺ redox cycling because iodide ions (I⁻) act as fast electron donors that readily reduce Fe³⁺ to Fe²⁺, forming reactive iodine radicals (I•). These iodine species are photo-active and further promote ROS generation under irradiation, creating a rapid and efficient Fe³⁺/Fe²⁺ regeneration loop. Unlike metal dopants, I⁻ reduces Fe³⁺ with lower energy barriers and without blocking active sites, resulting in faster redox turnover and higher oxidative efficiency^10^. After that, the samples were collected by centrifugation to separate the solid from the supernatant. The modified materials were thoroughly washed with ethanol and distilled water to remove any residues, as well as any by-products that may have formed during the process. The cleaned materials were then dried in two stages: initially at 70 °C for 3 h to eliminate surface moisture, followed by vacuum drying at 120 °C for 24 h to ensure complete removal of any entrapped solvents from the pores of the framework.

#### Solvent free synthesis of RTG-MIL-100(Fe) via KI-assisted

**Fig. 1:**
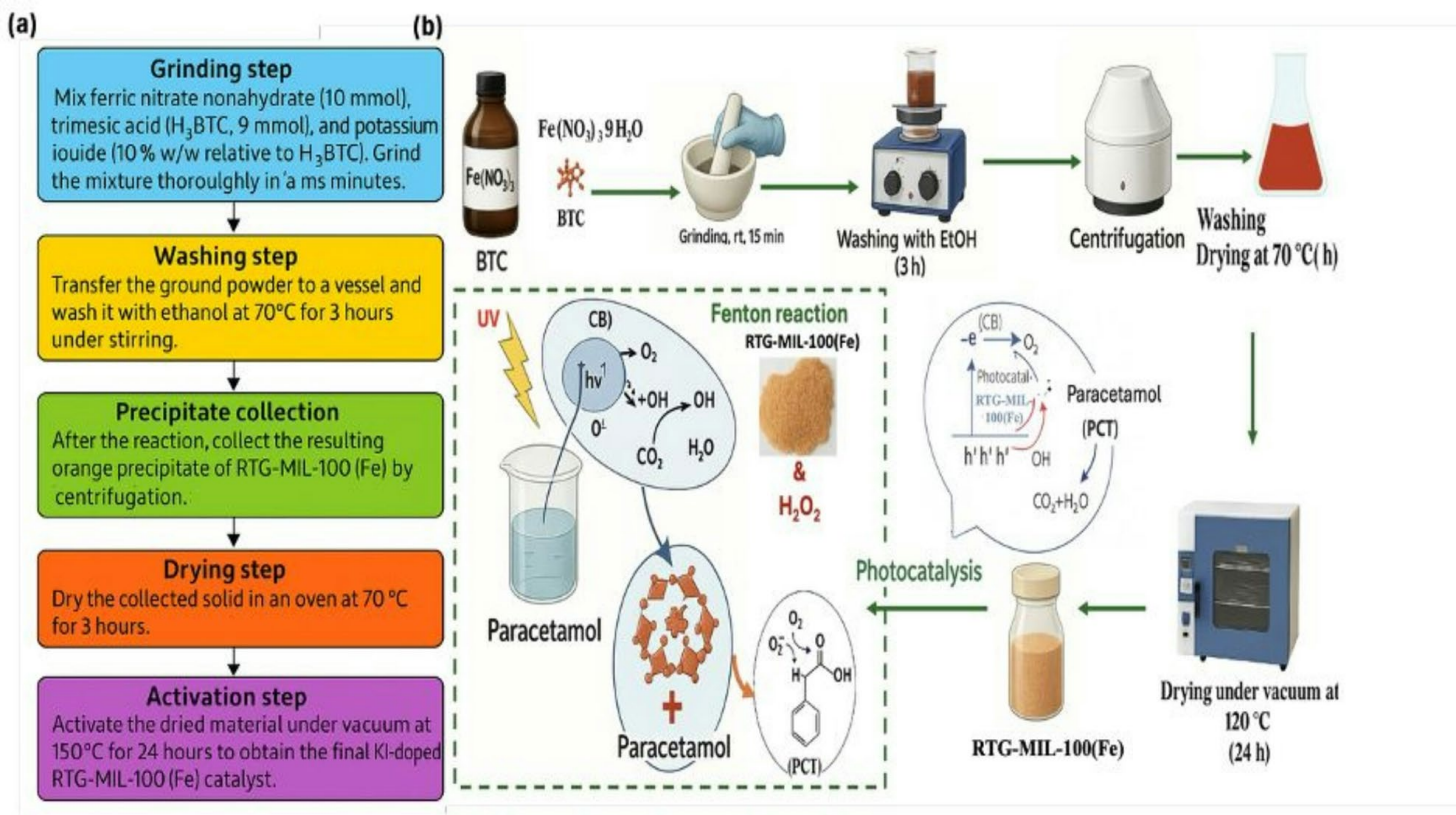
(**a**) Flow chart of the preparation of RTG-MIL-100(Fe) and (**b**) Graphical illustration of experimental treatment steps.

RTG-MIL-100(Fe) was synthesized by solvent free, room temperature synthesis. For clarity, a schematic flowchart has been included to visually summarize the step-by-step synthesis process (Fig. 1a). Firstly, ferric nitrate nonahydrate (10 mmol), trimesic acid (9 mmol) and 10% potassium iodide (10% based on H_3_BTC) were ground in a mortar for 15 min and then the powder was washed with ethanol at 70 °C for 3 h. After reaction, the resulting orange powder of RTG-MIL-100(Fe) as the main product was obtained. The precipitate was collected by centrifugation and dried in an oven at 70 °C for 3 h and activated under vacuum at 150 °C for 24 h.

### Characterization

Nitrogen adsorption–desorption isotherms were measured at − 196 °C using a 3 H-2000PS1 system after degassing the samples at 120 °C under vacuum for 2 h. XRD analysis was performed on a Siemens D500 diffractometer with Cu Kα radiation (λ = 0.15406 nm) over a 5–80° 2θ range. Surface morphology was examined by SEM (Zeiss Gemini Ultra, 5 kV, SE2 detector) and additional SEM images were captured with a SUPRA 55 (20 kV, Carl Zeiss AG). FT-IR spectra were recorded on a Perkin Elmer Spectrum 100 in the 400–4000 cm⁻¹ range. Metal leaching was quantified using inductively coupled plasma optical emission spectrometry (ICP-OES, Agilent 5100), performed according to APHA Standard Methods (20th ed., 2005). The precision of the measurements was ensured by triplicate analysis and calibration curves constructed for each metal ion. XPS was conducted using a Thermo Scientific Kα spectrometer with a monochromatic Al Kα source (1486.6 eV). Survey spectra were collected at 200 eV pass energy, and high-resolution spectra at 50 eV with step sizes of 0.4 eV and 0.1 eV, respectively.

The pHpzc of the RTG-MIL-100(Fe) catalyst was determined using the salt addition (pH-drift) method^39^. A series of 0.01 M NaCl solutions were prepared with initial pH values of 2, 4, 6, 8, and 10. The pH of each solution was adjusted using 0.1 M HCl or 0.1 M NaOH^40^. The catalyst suspensions were stirred for 24 h at room temperature, after which the final pH values were measured and compared to the initial values. The point at which the change in pH (ΔpH) becomes zero was taken as the pHpzc of the catalyst. When the solution pH is higher than the pHpzc, the catalyst surface becomes negatively charged, whereas at pH values lower than the pHpzc, the surface becomes positively charged. Plotting ΔpH versus the initial pH enabled accurate determination of the pHpzc.

### Wastewater treatment methodology

A 1000 ppm stock solution of PCT was prepared and diluted to the desired concentrations (5–40 ppm). For each experiment, 100 mL of the working solution was used for photocatalytic oxidation. The pH was adjusted to 3–7 using 0.1 M NaOH or 0.1 M H₂SO₄ and measured with a digital pH meter. The MOF catalyst (5–40 mg/L) was added to the solution and sonicated for 10 min to ensure proper dispersion, after which H₂O₂ (30 wt%) was introduced to initiate the Fenton reaction at concentrations ranging from 100 to 800 mg/L.

The mixture was irradiated with a low-pressure mercury UV lamp emitting at 287 nm with a power of 12 W, and samples were taken every 10 min. Before analysis, solutions were filtered through a 0.45 μm membrane to remove the catalyst. The residual PCT concentration was measured using a UV-Vis spectrophotometer at 243 nm. The oxidation efficiency was then calculated using Eq. (1):

1 $$\:\mathrm{P}\mathrm{C}\mathrm{T}\:\mathrm{r}\mathrm{e}\mathrm{m}\mathrm{o}\mathrm{v}\mathrm{a}\mathrm{l}\:\left(\mathrm{\%}\right)=\left(\frac{{C}_{0}-{C}_{t}}{{\mathrm{C}}_{0}}\right)*100$$

where, C_0_ and C are the concentrations of PCT at time *0* and *t*, respectively. The illustrated process is graphically represented in Fig. 1b.

### Radical scavenging activity test

Different scavengers were utilized to elucidate the nature of the reactive oxygen species (ROS) generated in the Fenton system. Initially, concentration of the scavengers, equal to 20 times the concentration of H_2_O_2_ used in the reaction system, was added to the reaction mixture. Specifically, 0.1 M isopropyl alcohol (IPA) was used as a hydroxyl radical (^**⋅**^OH) scavenger, 0.1 M ammonium oxalate (AO) as a hole (h⁺) scavenger, and 0.1 M silver nitrate (AgNO_3_) as an electron (e⁻) scavenger. Each was added separately to 100 mL of a 10 ppm PCT solution. Next, the suspensions were stirred for 15 min in the dark to ensure adsorption–desorption equilibrium before adding 400 ppm of 30% H₂O₂. The photocatalytic reaction was then initiated under UV irradiation, and aliquots were collected at regular time intervals for analysis.

## Results and discussion

### Structural integrity

**Fig. 2:**
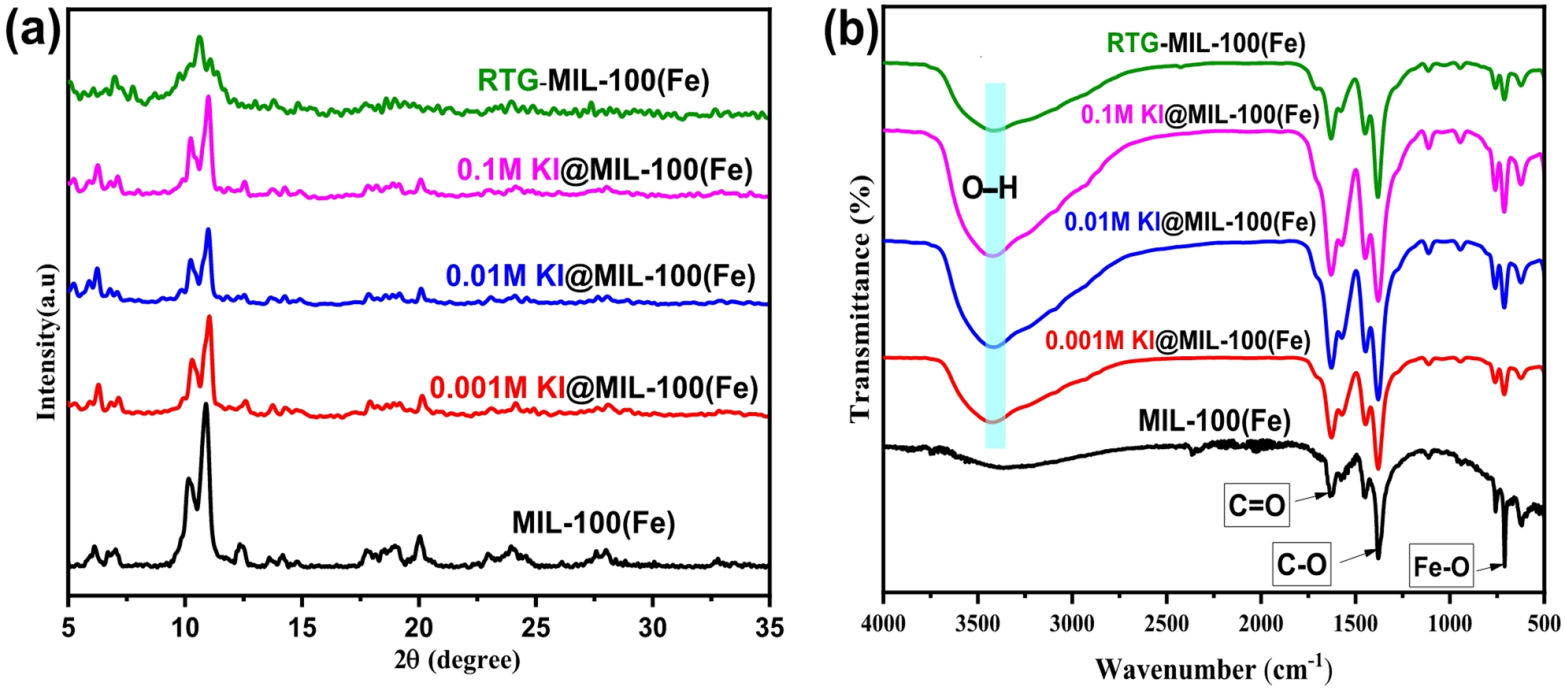
(**a**) XRD patterns of MIL-100(Fe), xM KI@ MIL-100(Fe) (x = 0.1 M, 0.01 M, 0.001 M), RTG-MIL-100(Fe) samples and (**b**) FTIR spectra illustrating the functional groups of these samples.

Figure 2a displays X- Ray Diffraction (XRD) of RTG-MIL-100(Fe)and xM KI@MIL-100(Fe) (x = 0.1, 0.01, 0.001 M). XRD was used to confirm the crystalline structure and phase purity of the MOF, with the characteristic diffraction peaks verifying the successful formation of the MIL-100(Fe) framework. Pristine MIL-100(Fe) was used as a reference, showing characteristic peaks at 2θ of 10.3° and 13.1°, confirming framework formation^27^. Both 0.001 M and 0.01 M KI-modified samples retained these reflections, indicating the structure was preserved at low iodide concentrations. A reduction in peak intensity was observed, suggesting decreased crystallinity or local distortion due to iodide–Fe–O interactions. At 0.1 M KI, the peak intensity further decreased, implying increased disorder and partial amorphization. In contrast, the RTG-MIL-100(Fe)sample exhibited broad, weak peaks, confirming high defect density and poor crystallinity. No new crystalline KI phases were detected, indicating high KI dispersion within the MOF, with low KI loadings maintaining framework integrity and high KI loading inducing defects that may enhance photocatalytic activity.

Figure 2b shows Fourier Transforms Infrared Spectroscopy (FT-IR) of samples. FT-IR spectroscopy was utilized to identify functional groups and evaluate Fe–O and carboxylate coordination modes, offering additional confirmation of framework formation and potential structural changes. All samples exhibit characteristic vibrational bands of the MIL-100(Fe) framework, confirming the successful formation of the MOF structure. Additionally, the intense band in the region of 1700–1600 cm⁻¹ corresponds to the asymmetric stretching vibration of C = O in the carboxylate groups from the organic linker (H_3_BTC), while bands around 1400–1300 cm⁻¹ are due to symmetric C–O stretching. The peaks near 750–600 cm⁻¹ are attributed to Fe–O stretching, indicating the presence of Fe_₃_O metal clusters which confirms the presence of metal-oxygen bonding within the MOF structure^41,42^. A broad absorption band centered around 3400 cm⁻¹, attributed to the O–H stretching vibrations of surface hydroxyl groups and/or adsorbed water molecules, was observed in all samples. This band appeared more intense in the modified samples compared to the pristine hydrothermal MIL-100(Fe), suggesting a higher content of surface –OH groups or retained moisture. It is noteworthily to mention that an increased concentration of hydroxyl groups is advantageous for photocatalytic applications, as these groups can enhance surface hydrophilicity and facilitate the formation of reactive hydroxyl radicals (^•^OH) under light irradiation, ultimately leading to improved oxidation efficiency of organic pollutants^43^.

**Fig. 3:**
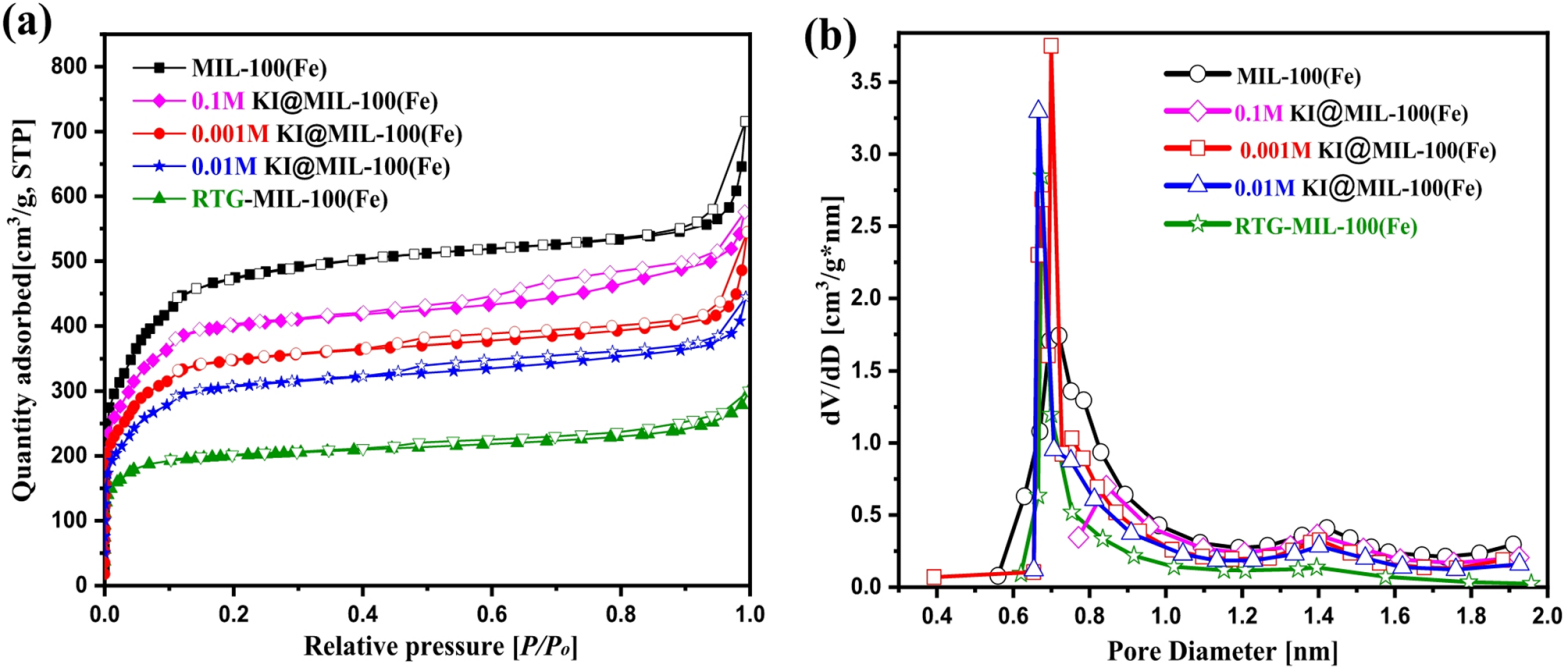
(**a**) BET analysis of MIL-100(Fe), xM KI@MIL-100(Fe) (x = 0.1 M, 0.01 M, 0.001 M), RTG-MIL-100(Fe)at 77 K and (**b**) pore size distribution of samples.

The N₂ adsorption–desorption isotherms (Fig. 3a) of MIL-100(Fe) and its KI-modified counterparts clearly demonstrate the impact of iodide incorporation on the textural properties of the framework. BET surface area analysis was carried out to assess the material’s porosity and specific surface area, which are critical parameters governing adsorption and catalytic performance in photo-Fenton-like systems. Pristine MIL-100(Fe) exhibited the highest N₂ uptake with BET surface area 1805 m^2^/g and pore volume 0.69 cm^3^/g, as summerized in Table 1, reflecting its intrinsically high surface area and hierarchical micro–mesoporous nature^44^. After KI modification, a systematic decrease in the adsorption capacity was observed, with the extent of reduction correlating with the concentration of KI introduced. Specifically, 0.1 M KI@MIL-100(Fe) retained a moderate uptake with BET surface area 1117 m^2^/g and pore volume 0.6 cm^3^/g, while further decreases were recorded for the 0.001 M and 0.01 M KI@MIL-100(Fe) samples (~ 999–1048 m^2^/g). The most pronounced reduction occurred for RTG-MIL-100(Fe), which displayed a drastically lower uptake with BET surface area 778 m^2^/g and pore volume 0.3 cm^3^/g, indicative of severe pore blocking or partial pore filling by KI species. Despite the decrease in capacity, all isotherms preserved the characteristic Type I profiles with steep uptakes at low relative pressures and slight hysteresis at higher pressures, consistent with micro–mesoporous architectures.

The pore size distribution curves (Fig. 3b) confirm the observed textural changes. Pristine MIL-100(Fe) shows a sharp peak at ~ 0.8 nm, characteristic of its microporous cages. With KI modification, this peak intensity decreases, indicating reduced pore volume due to partial blockage by iodide/iodine species. Notably, the pore size itself remains unchanged, confirming that the framework integrity is preserved. Thus, the decline in adsorption arises from pore occupation rather than structural collapse. KI incorporation effectively lowers surface area and pore volume while maintaining stability. This modification may hinder reactant diffusion but simultaneously introduce iodide functionalities that enhance Fenton-like activity. Hence, balancing textural loss with catalytic gain is crucial in designing iodide-modified MIL-100(Fe).

**Table 1:** Textural properties of various samples.

MOF Types	BET Surface Area (m^2^/g)	Langmuir Surface Area (m^2^/g)	Pore Volume (cm^3^/g)	Pore Diameter (nm)
MIL-100(Fe)	1805	2312	0.69	2.45
RTG- MIL-100(Fe)	778	918	0.30	2.26
0.1 M KI-MIL-100(Fe)	1117	1884	0.60	2.75
0.01 M KI-MIL-100(Fe)	999	1455	0.48	2.71
0.001 M KI-MIL-100(Fe)	1048	1635	0.52	2.78

**Fig. 4:**
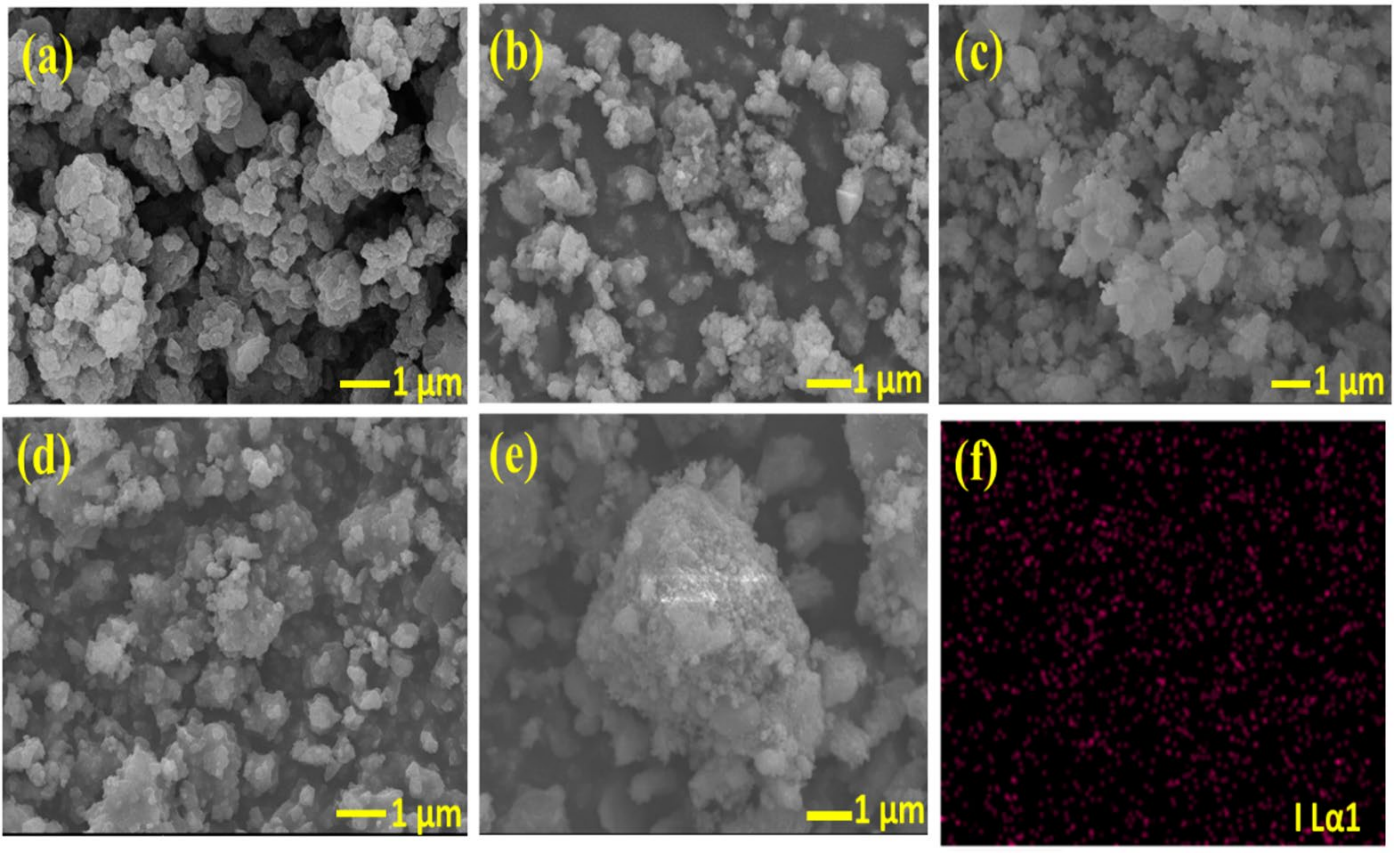
SEM images of (**a**) MIL-100(Fe) (**b**) 0.1MKI@MIL-100(Fe) (**c**) 0.01MKI@MIL-100(Fe) (**d**) 0.001MKI@MIL-100(Fe), (**e**) RTG-MIL-100(Fe), and (**f**) EDX Elemental Mapping.

The SEM images in Fig. 4 reveal the visual insights into particle morphology, size distribution, and surface texture, revealing the uniformity and structural integrity of MIL-100(Fe) and its KI-modified derivatives. Pristine MIL-100(Fe) (Fig. 4a) displays well-defined polyhedral crystals with relatively uniform size distribution, indicating the successful formation of the framework^44^. Upon KI modifications, the morphology becomes less ordered, as observed in 0.1 M KI@MIL-100(Fe) (Fig. 4b), where smaller fragmented particles are distributed on the surface, suggesting partial structural distortion. The 0.01 M KI@MIL-100(Fe) sample (Fig. 4c) maintains some crystalline features, but with reduced particle size and rougher surfaces compared to the parent MIL-100(Fe). In the case of 0.001 M KI@MIL-100(Fe) (Fig. 4d), the particles appear more agglomerated with less distinct crystal edges, highlighting the influence of iodide on crystal growth. The RTG-MIL-100(Fe)sample (Fig. 4e) exhibits large irregular aggregates and significant collapse of the original morphology, indicating excessive KI loading disrupts framework crystallinity. Overall, the SEM observations confirm that KI incorporation progressively alters the morphology of MIL-100(Fe), from ordered polyhedral crystals to agglomerated and irregular particles at higher KI content. EDX elemental mapping (Fig. 4f) shows weak, sparsely distributed iodine signals, indicating that only small amounts of iodide remain within the MIL-100(Fe) framework. This finding is consistent with the XPS results, where the low-intensity I 3d peaks confirm the presence of trace iodide species after KI modification.

**Fig. 5:**
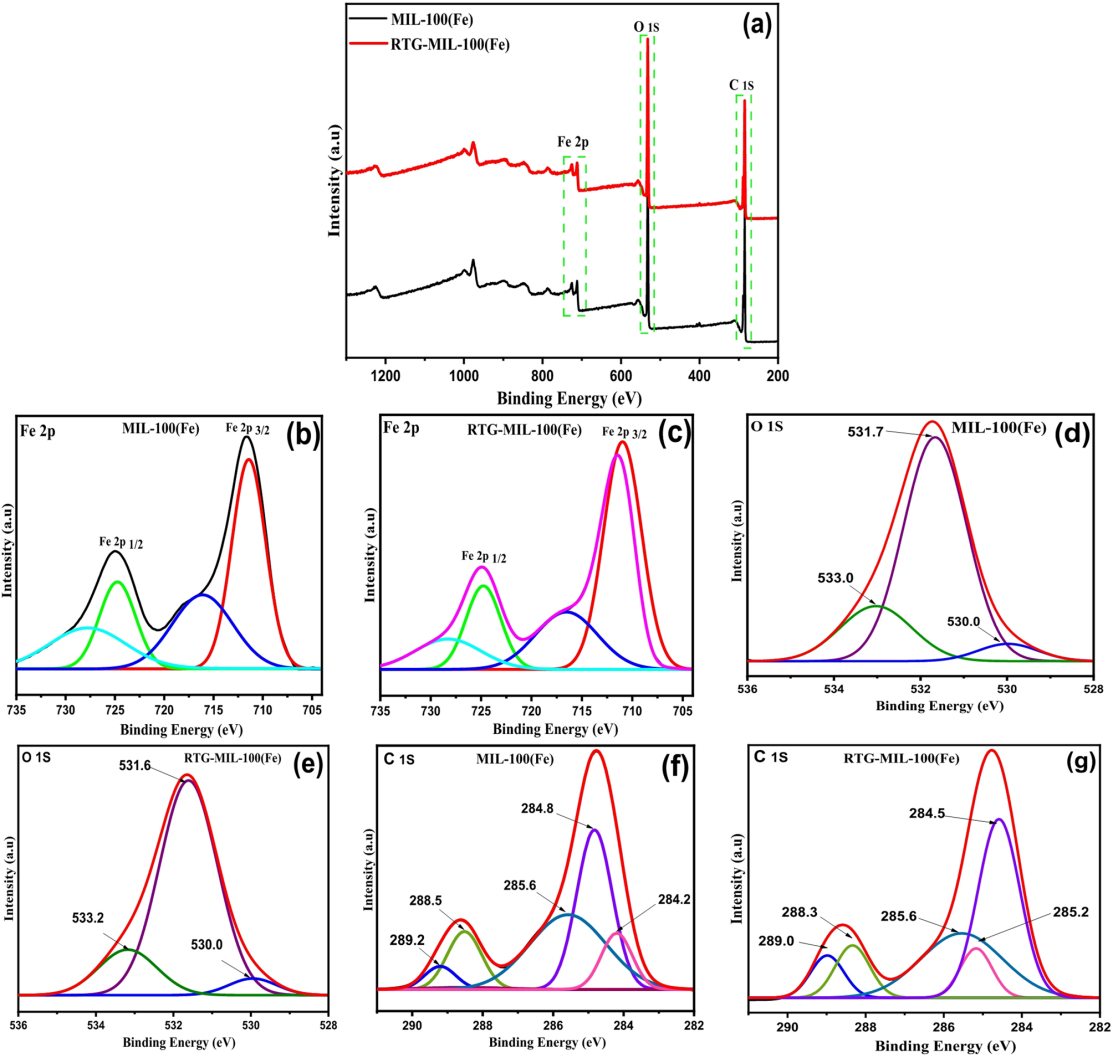
XPS spectra of MIL-100(Fe) and RTG-MIL-100(Fe); (**a**) XPS survey (**b**,** c**) Fe 2p scan (**d**,** e**) O 1s, and (**f**,** g**) C 1s spectra for MIL-100(Fe) and RTG-MIL-100(Fe).

The XPS analysis was carried out to determine the surface chemical states of iron and to quantify the Fe³⁺/Fe²⁺ ratio, providing essential information about the redox behavior that drives the photo-Fenton-like mechanism. The chemical states of Fe, O, and C in MIL-100(Fe) and KI-modified MIL-100(Fe) and RTG-MIL-100(Fe), were investigated to elucidate the role of KI in tuning the redox properties of the catalyst. The wide survey spectra (Fig. 5a) confirmed the presence of Fe, O, and C elements in both samples, consistent with the MIL-100(Fe) framework, while the KI-modified sample showed slight changes in peak position and intensity, suggesting chemical interaction between iodide ions and the Fe–O Clusters.

The Fe 2p spectra provided direct evidence of redox modifications induced by iodide incorporation. In pristine MIL-100(Fe), the Fe 2p spectrum exhibited the characteristic peaks at ~ 711 eV (Fe 2p₃/₂) and ~ 724 eV (Fe 2p₁/₂), along with satellite features, confirming the predominance of Fe³⁺ species in the framework (Fig. 5b). In contrast, the KI-modified MIL-100(Fe) sample displayed a clear shift of the Fe 2p₃/₂ peak toward lower binding energies and an increased contribution from Fe²⁺, as revealed by peak deconvolution. The Fe 2p peak deconvolution were fitted using a Gaussian–Lorentzian mixed function, yielding low residuals and a high goodness of fit, which confirms the reliability of separating Fe²⁺ and Fe³⁺ components. The KI-modified sample exhibits a clearly increased Fe²⁺ contribution, evidenced by the enlarged area of the low–binding-energy Fe²⁺ peaks. This enhancement in the Fe²⁺ fraction is consistent with improved Fe³⁺/Fe²⁺ redox cycling and directly explains the superior catalytic performance of the KI-modified MIL-100(Fe). This indicates that iodide ions acted as reducing agents, donating electrons to partially reduce Fe³⁺ to Fe²⁺ within the framework (Fig. 5c). Such Fe³⁺/Fe²⁺ redox adjustment is crucial for enhancing catalytic activity, as Fe²⁺ sites are more effective in decomposing H₂O₂ to generate reactive hydroxyl radicals in the Fenton-like process. The results clearly reveal that the incorporation of KI significantly increases the Fe²⁺ fraction, shifting the Fe²⁺/Fe³⁺ ratio from 0.47 in pristine MIL-100(Fe) to 1.22 in RTG-MIL-100(Fe). This higher Fe²⁺/Fe³⁺ ratio Confirms that iodide acts as an electron donor, effectively reducing Fe³⁺ to Fe²⁺, and thus enhancing the redox cycling efficiency during the Fenton-like reaction. This XPS-derived increase in the Fe²⁺ fraction is fully consistent with the catalytic results. RTG-MIL-100(Fe) exhibits the highest pseudo-first-order rate constant for PCT degradation and achieves almost complete PCT removal (99.6%) under the optimized conditions. This clear correlation between the higher Fe²⁺/Fe³⁺ ratio and the enhanced degradation rate confirms that the iodide-induced redox modulation revealed by XPS is directly responsible for the superior photo-Fenton-like performance of RTG-MIL-100(Fe). The O 1s spectra further supported this observation (Figs. 5d and e). MIL-100(Fe) exhibited three main components corresponding to lattice oxygen (~ 530.0 eV), Fe–O bonds (~ 531.7 eV), and surface hydroxyl or adsorbed oxygen species (~ 533.0 eV). In the KI-modified sample, the relative intensity of the lattice oxygen peak decreased, accompanied by a higher contribution from surface oxygen-related species. This suggests that KI incorporation induces local distortions in the Fe–O Coordination environment and creates oxygen vacancies, which can further promote redox cycling and catalytic activity. The C 1s spectra of both samples were dominated by peaks corresponding to C–C/C = C (~ 284.5 eV), C–O (~ 285.6 eV), and O–C = O (~ 288.5–289.0 eV), indicating that the organic linker backbone of MIL-100(Fe) remained intact after KI modification (Figs. 5f and g). This confirms that the structural integrity of the framework is maintained, while its electronic environment is selectively altered.

Overall, the XPS results clearly demonstrate that the incorporation of KI plays a pivotal role in enhancing the redox chemistry of MIL-100(Fe). The introduction of iodide ions partially reduces Fe³⁺ to Fe²⁺ and creates surface oxygen defects, leading to a higher Fe²⁺/Fe³⁺ ratio and improved active sites for H₂O₂ activation. These modifications directly explain the superior catalytic activity of RTG-MIL-100(Fe) observed in the photo-Fenton oxidation of PCT, as the enhanced Fe³⁺/Fe²⁺ redox cycling accelerates hydroxyl radical generation under UV-assisted conditions.

Compared with other dopant species, iodide exhibits a comparatively low redox potential (I₂/I⁻ = +0.54 V)^45^, making it a highly effective electron mediator in Fenton-like systems. This favorable potential enables I⁻ to readily donate electrons to Fe³⁺, driving its rapid reduction to Fe²⁺ through a thermodynamically preferred pathway. The resulting iodine radicals (I•/I₂•⁻) formed during this process are not only stable enough to participate in secondary redox events but also photo-reactive, allowing them to further promote ROS formation under irradiation. Consequently, I⁻ functions as a dual-purpose redox shuttle that is signified as chemically reducing Fe³⁺ while simultaneously enhancing photochemical activation of H₂O₂. This synergistic behavior results in a significantly faster and more sustained Fe³⁺/Fe²⁺ regeneration cycle than what is typically achieved with conventional metal dopants or heteroatom modifications, which lack this low-energy electron-transfer pathway and do not generate photoreactive intermediates. Hence, KI doping provides a uniquely efficient mechanism for maintaining high catalytic turnover during the photo-Fenton process.

### Catalytic oxidation of PCT wastewater

#### Comparison of various oxidation systems reaction time

**Fig. 6:**
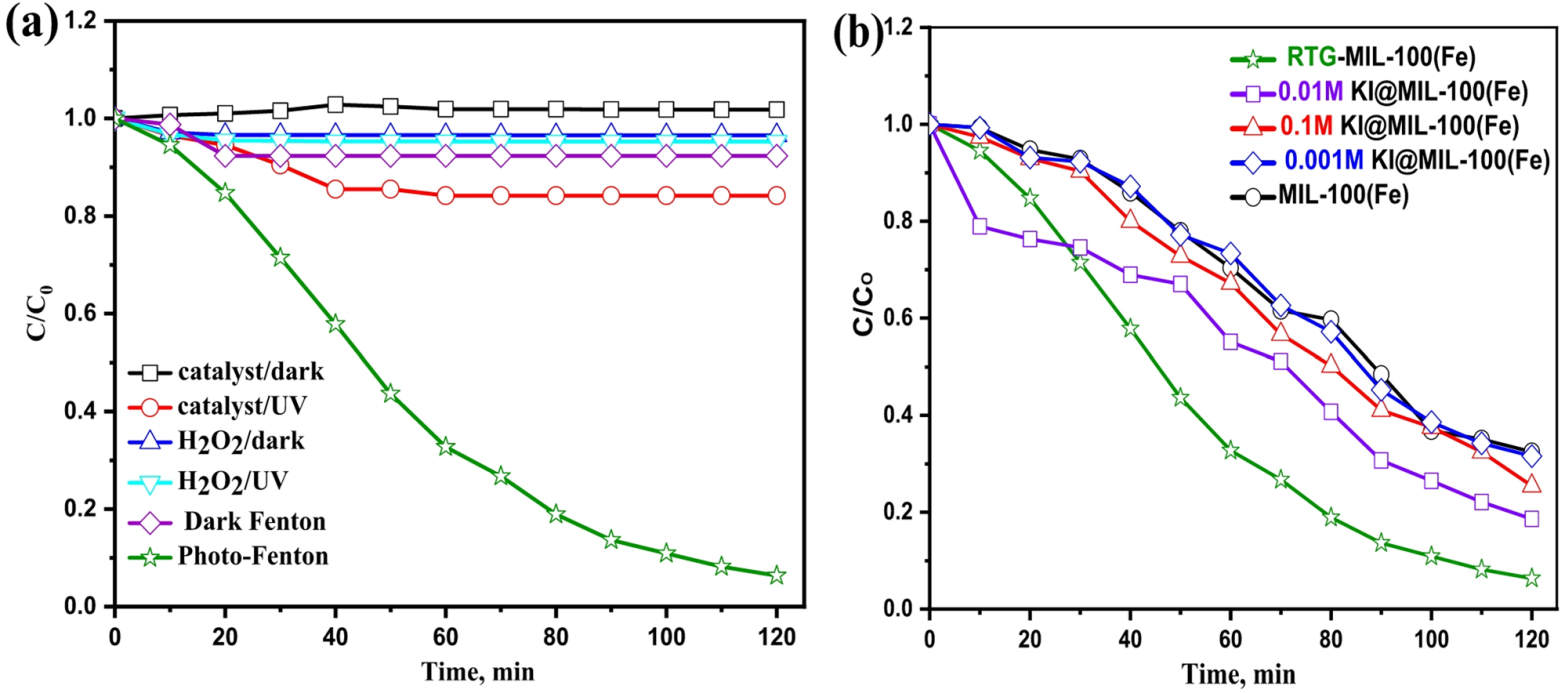
Effect of PCT removal (**a**) using various catalytic systems and (**b**) comparison between different MOFs based Photo-Fenton-like oxidation.

The oxidation behavior of PCT under different treatment systems was examined (Fig. 6a). At pH 5.5 with 40 mg/L catalyst and 400 ppm H₂O₂, only minimal oxidation occurred in the absence of UV or H₂O₂, confirming that adsorption or direct photolysis alone is insufficient. Dark conditions with catalyst or H₂O₂ showed negligible removal (< 7%). Moderate oxidation (about 20% removal) was observed when catalysts were combined with UV light, indicating Fe-based catalytic activity in electron generation^46^. The highest efficiency was achieved with the photo-Fenton process, where UV, H₂O₂, and catalyst synergistically produced hydroxyl radicals, leading to rapid PCT degradation. Removal efficiencies followed the order: MIL-100(Fe) (67.5%) < 0.1 M KI@MIL-100(Fe) (74.6%) < 0.01 M KI@MIL-100(Fe) (81.4%) < 0.001 M KI@MIL-100(Fe) (68.4%) < RTG-MIL-100(Fe) (93.6%) as exhibited in Fig. 6b. The superior activity of RTG-MIL-100(Fe)is attributed to an enhanced Fe³⁺/Fe²⁺ ratio, improving H₂O₂ activation and confirming the effectiveness of heterogeneous photo-Fenton-like systems.

#### Optimized the catalytic activity parameters

Among the synthesized materials, RTG-MIL-100(Fe) was identified as the most promising catalyst due to two key advantages: (i) it demonstrated the highest photocatalytic efficiency at the natural solution pH (Fig. 6b), eliminating the need for pH adjustment, and (ii) it was synthesized through a simple, energy-efficient, room-temperature method, offering a greener alternative to Conventional hydrothermal or post-synthetic approaches. To further understand its superior activity, a systematic study was performed by varying operational parameters, including solution pH, catalyst dosage, initial pollutant concentration, and H₂O₂ dosage, to optimize the photocatalytic performance.

##### Effect of pH in natural wastewater

**Fig. 7:**
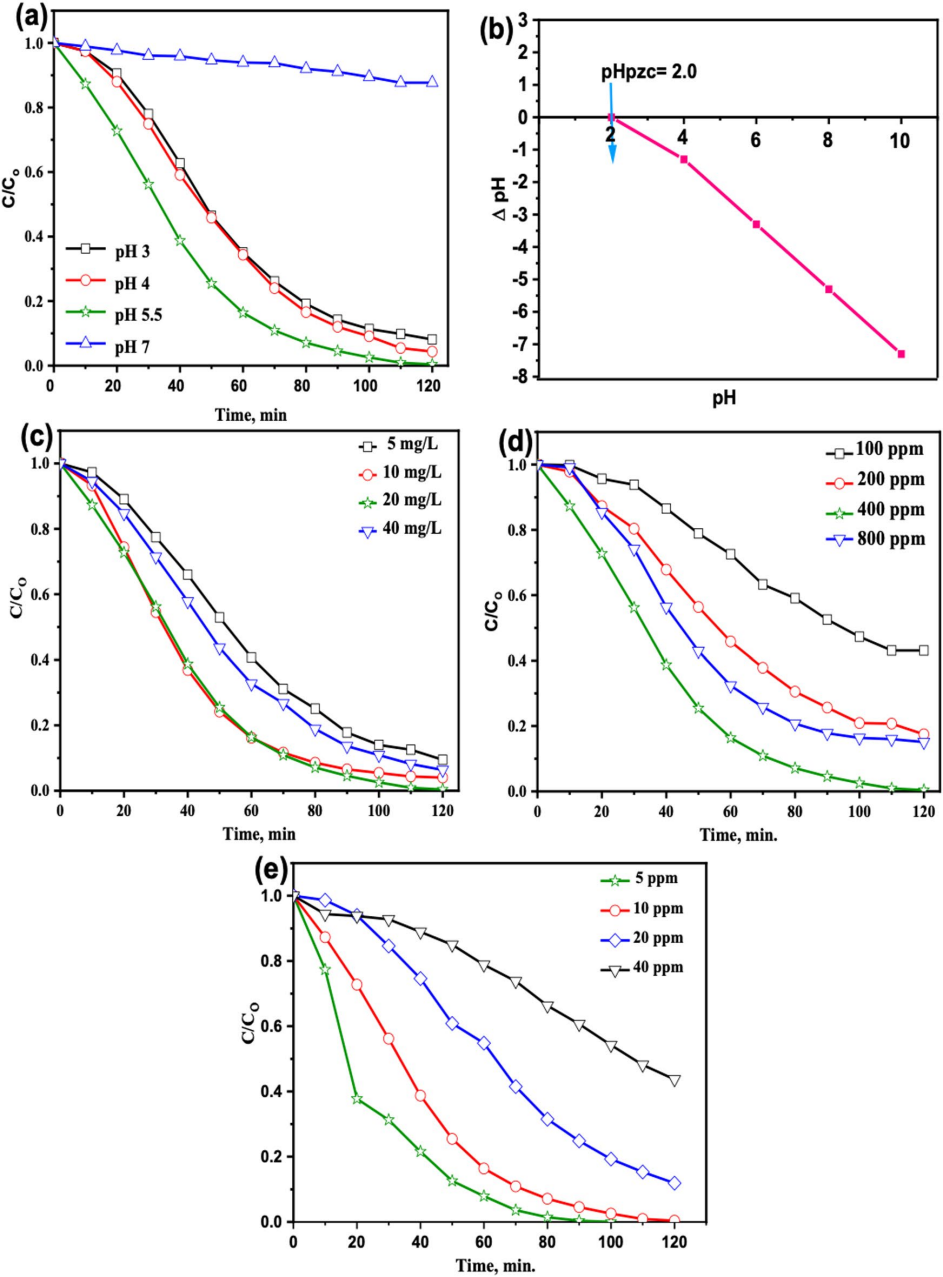
Effect of different oxidation parameters on PCT oxidation: (**a**) Effect of solution pH (PCT 10 mg/L, H₂O₂ 400 ppm, catalyst 20 mg/L) and the (**b**) Point of Zero charge of RTG-MIL-100(Fe); (**c**) Effect of RTG-MIL-100(Fe) catalyst dose (PCT 10 mg/L, H₂O₂ 400 ppm, pH 5.5); (**d**) Effect of H₂O₂ dosage (PCT 10 mg/L, catalyst 20 mg/L, pH 5.5) and (**e**) Effect of initial PCT concentration in the Fenton system (pH 5.5, catalyst 20 mg/L, H₂O₂ 400 ppm).

In Fenton systems, the pH of the reaction medium plays a crucial role in governing the overall oxidation efficiency. This is primarily due to its influence on the redox interaction between H₂O₂ and iron ions, which ultimately dictates the formation rate of hydroxyl radicals. As shown in Fig. 7a, PCT removal exhibited a strong pH dependence, reaching a maximum efficiency of 99.6% at pH 5.5 (the original solution pH). The efficiency decreased to 95.6% at pH 4, 91.9% at pH 3, and dropped sharply to 12.3% under near-neutral conditions (pH 7).

This behavior can be rationalized by considering the catalyst surface charge, the ionization state of PCT, and the pH-sensitive reactivity of H₂O₂. The pH at the point of zero charge (pHpzc) of RTG-MIL-100(Fe) was determined to be approximately 2.0 (Fig. 7b). Thus, at pH values above the pHpzc that including pH 5.5, which the catalyst surface becomes negatively charged. Although electrostatic attraction toward PCT (pKa 9.5, predominantly neutral at pH 5.5) is not expected under these conditions, high degradation efficiency was still achieved. This is attributed to the accelerated Fe³⁺/Fe²⁺ redox cycling enabled by KI modification. Moreover, the system exhibits enhanced activation of H₂O₂ at mildly acidic pH, with •OH radicals predominantly generated in the bulk solution rather than through surface-controlled pathways. At lower pH values (pH 3–4), the stronger acidity facilitates the reduction of Fe³⁺ to Fe²⁺, thereby enhancing the Fenton-like catalytic cycle. However, the slightly reduced removal efficiency relative to pH 5.5 may be associated with protonation of active sites or partial degradation of the MOF framework under harsh acidic conditions^47^. Additionally, excess H⁺ ions can scavenge •OH radicals, lowering the effective oxidative capacity of the system.

In contrast, at near-neutral pH (pH 7), the degradation efficiency declined markedly to 12.3%. This can be attributed to a reduction in Fe²⁺ availability, the diminished decomposition rate of H₂O₂, and the limited formation of •OH radicals under such conditions. Moreover, iron-based catalysts typically exhibit deactivation at higher pH due to iron hydroxide precipitation or surface passivation^48^. Hence, these results verify that mildly acidic conditions especially pH 5.5, which offer an optimal balance of catalyst stability, effective Fe³⁺/Fe²⁺ cycling, and efficient ROS generation during the Fenton-like oxidation of PCT using RTG-MIL-100(Fe)^49^.

##### Effect of MOF-catalyst amount on PCT oxidation

To determine the optimal catalyst loading for the MOF based Fenton-like oxidation system, a series of experiments were conducted using by varying the concentrations of RTG-MIL-100(Fe)(5, 10, 20, and 40 mg/L), while keeping all other parameters are kept constant (10 ppm PCT, 400 ppm H₂O₂, and pH 5.5). As presented in Fig. 7c, the highest removal efficiency (99.6%) was achieved at a catalyst dose of 20 mg/L, indicating it as optimal catalyst dose. At this dose, the amount of reactive species (e.g., •OH radicals) generated is sufficient to effectively degrade PCT^50^. However, further increase in catalyst dosages beyond 20 mg/L led to a reduction in oxidation efficiency. This decline is likely due to Catalyst particle aggregation at higher catalyst loading, which reduces the available surface area and active sites, and limits light penetration in photocatalytic environments. As a result, catalytic activity diminishes, thereby lowering the oxidation rate and leading to a less efficient Fenton-like process. These findings are consistent with previously reported studies, which also observed similar trends at higher catalyst loadings^51^.

##### Effect of H_2_O_2_ dosage

Hydrogen peroxide concentration is a critical parameter in Fenton-like systems, as it directly influences the generation of hydroxyl radicals(•OH), which are the main oxidative agents. To determine the optimal H₂O₂ dose for efficient PCT oxidation, experiments were conducted under fixed conditions: pH 5.5, 10 ppm PCT, and 20 mg/L of RTG-MIL-100(Fe)catalyst. The effect of increasing H₂O₂ concentration on the oxidation efficiency is illustrated in Fig. 7d. The study explored a range of H₂O₂ concentrations from 100 to 800 ppm. As the H₂O₂ dose increased from 100 to 400 ppm, the oxidation efficiency significantly improved, with removal percentages of 56.9%, 82.5%, and 99.6% for 100, 200, and 400 ppm, respectively. This enhancement is attributed to the increased availability of •OH radicals generated from the decomposition of H₂O₂ in the presence of the Fe-based catalyst^52^. However, further increase in the H₂O₂ concentration to 800 ppm resulted in a slight decrease in removal efficiency to 84.9%. This decline can be explained by the scavenging effect, where excess H₂O₂ begins to Consume the generated •OH radicals through side reactions (see Eqs. 2 and 3), thus lowering the effective radical concentration available for PCT oxidation^53^. Consequently, while a moderate increase in H₂O₂ improves the oxidative performance, excessive concentrations may lead to self-quenching effects and reduced overall efficiency. Therefore, 400 ppm was identified as the optimal H₂O₂ dose under the given conditions.

2 $$\:{{H}_{2}{O}_{2}+\:}^{.}OH\:\to\:\:{\:\:}^{.}O{H}_{2}+{H}_{2}O$$

3 $$\:{\:\:}^{.}O{H}_{2}+{{\:}^{.}OH\to\:H}_{2}O+{O}_{2}$$

##### Effect of initial PCT concentration

To emphasize the practical applicability of real systems, the effect of emerging contaminant (PCT) different initial concentration on the photocatalytic oxidation rate was conducted in concentration range from 5 to 40 ppm of the solution using 400 ppm of H_2_O_2_, 20 mg/L of a catalyst load at original pH (5.5). Figure 7e shows that as the PCT solution becomes more concentrated, the photocatalytic oxidation rate tends to decrease. This indicates that there should be a maximum PCT distribution allowing for a successful reaction at active sites. In addition, the emerging contaminant PCT is inefficiently adsorbed to avoid encounters with UV photons, resulting in a reduction in the photooxidation rate^54^. Although the probability of photocatalyst excitation decreased at higher concentration levels, the screening effect also predominates, and hence the oxidation rate decreased^51^. Figure 7e reveals that RTG-MIL-100(Fe) demonstrated a complete removal of the emerging contaminant PCT at the concentration of 5 ppm within 100 min. Notably, RTG-MIL-100(Fe) performed effective removal of the emerging contaminant PCT at the concentration of 10 ppm and achieved an almost complete removal 99.6%.

### Thermodynamic and kinetic studies

**Fig. 8:**
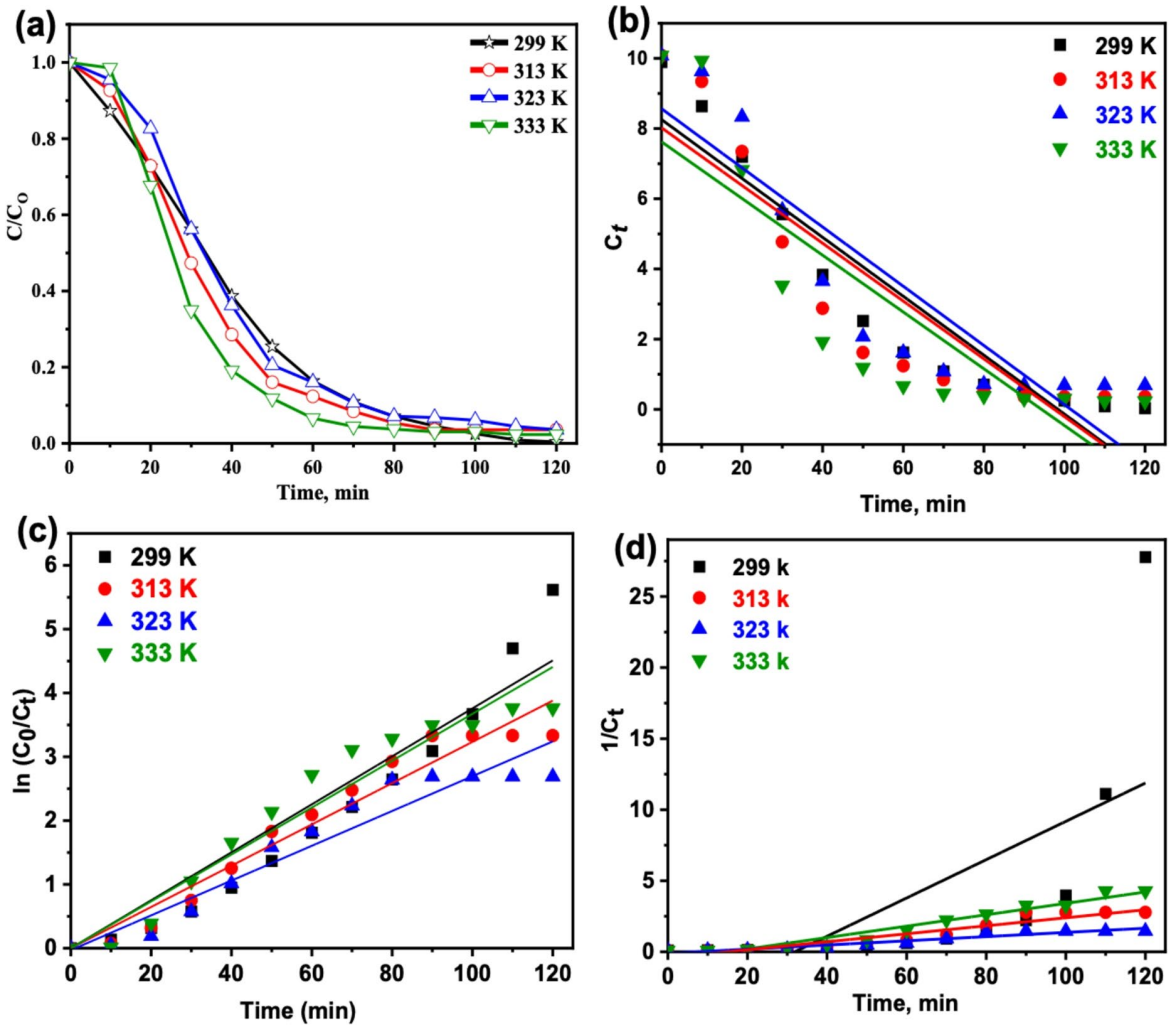
(**a**) Effect of temperature on PCT oxidation (PCT 10 mg/L, RTG-MIL-100(Fe) catalyst 20 mg/L, 400 ppm H_2_O_2_, and pH 5.5) and the corresponding kinetic models of (**b**) zero, (**c**) first and (**d**) second order plots.

The effect of temperature on the Fenton oxidation of paracetamol using RTG-MIL-100(Fe)was evaluated at 301–333 K (Fig. 8a). At room temperature (301 K), the catalyst achieved 99.6% removal, confirming highly efficient performance under ambient conditions. Slightly lower efficiencies were observed at 313 K (96.4%) and 323 K (93.2%), while a minor increase was noted at 333 K (97.7%). This trend reflects the dual role of temperature in Fenton systems: moderate heating can enhance •OH generation by accelerating molecular collisions, whereas excessive heating promotes H₂O₂ decomposition to O₂ and H₂O, reducing radical availability. Higher temperatures may also increase •OH scavenging via side reactions^55^. Overall, the results highlight that thermal activation is unnecessary, as the catalyst exhibits excellent activity at room temperature, underscoring its economic and practical advantages^46,47^.

To reach real world application, it is essential to study kinetic investigation for design aspects. In this regard, the kinetic studies were conducted for the synthesized RTG-MIL-100(Fe) sample for the oxidation of PCT in the presence of H₂O₂^56^. Kinetic studies are examined as a function of time and the data is fitted in the linearized integrated form of equations for zero, first and second order kinetic models and exhibited in Figs. 8 (b-d). The rate constants are then investigated by calculations based on linearized form of equations. The common three kinetic models of zero, first, second-order models are applied in their linearized forms according to the following equations^57^:

4 $$\:{C}_{t}={C}_{o}-{k}_{0}t$$

5 $$\:{C}_{t}={C}_{o}-{e}^{{k}_{1}t}$$

6 $$\:\left(\frac{1}{{C}_{t}}\right)=\left(\frac{1}{{C}_{0}}\right)-{k}_{2}t$$

Where *C*_*o*_ and *C*_*t*_ are the concentration at the initial oxidation time and time *t*, respectively. Also, *k*_*0*_, *k*_*1*_, *k*_*2*_ kinetic rate constants of zero-, first- and second-reaction kinetic models, respectively. While *R*^*2*^ is the correlation coefficient and *t*_*1/2*_ is the half-life reaction time. The correlation coefficient value was also applied to assess such compared models. Comparing *R*^*2*^ values estimated from the plot of such equations suggesting the best model fitted. The linear $\:\:ln(C₀/Cₜ)$versus time plots at different temperatures (Fig. 8b) showed excellent linearity (R² = 0.92–0.95), confirming that PCT degradation follows a pseudo-first-order model. Although the k₁ values in Table 2 do not increase monotonically with temperature, this slight variation is common in heterogeneous Fenton systems and is mainly attributed to changes in adsorption–desorption equilibrium and partial mass-transfer limitations at higher temperatures. These effects may temporarily offset the intrinsic Arrhenius increase. Nevertheless, the consistently high R² values and the close k₁ range strongly validate the pseudo-first-order kinetics.

**Table 2:** Kinetic model parameters for the photo-Fenton-like oxidation of PCT using the RTG-MIL-100(Fe)/H₂O₂/UV system.

Temp. K	Zero-order model			First-order model			Second-order model		
	k_0_ (min^-1^) ×10^2^	t_1/2_ (min)	*R* ^2^	k_1_(min^-1^) ×10^2^	t_1/2_(min)	*R* ^2^	k_2_ (min^-1^) ×10^2^	t_1/2_(min)	*R* ^2^
301	8.39	58.97	0.88	4.49	15.43	0.95	1.48	6.81	0.80
313	8.23	61.21	0.79	3.30	20.99	0.95	2.00	4.97	0.83
323	8.43	59.75	0.81	3.07	25.42	0.92	1.49	6.67	0.83
333	8.08	62.34	0.72	3.58	19.31	0.94	3.30	3.00	0.87

To extra assess the MOF based Fenton reaction treating PCT, thermodynamic parameters were evaluated and the data recorded in Table 3. The thermodynamic activation parameters were determined using the Arrhenius equation, based on the first-order kinetic model, as described in (Eq. 7).

7 $$\:{ln}{k}_{1}={ln}A-\frac{{E}_{a}}{RT}$$

where *A* is the pre-exponential factor constant; *E*_*a*_ is the activation energy (kJ mol^− 1^); *R* is the gas constant (8.314 J mol^− 1^ K^− 1^) and *T* is temperature (K). Therefore, *E*_*a*_ can be estimated from the linear plot of -ln*k*_*2*_ versus *1/T*. Therefore, the energy of activation value is estimated as 6.27 kJ.mol^− 1^. This minimal value of *E*_*a*_ confirmed the oxidation of PCT through modified MOF based Fenton reaction typically occurs at low energy barrier. Furthermore, the thermodynamic activation parameters associated with the PCT removal process were evaluated using the Eyring equation (Eq. 8).

8 $$\:{k}_{1}=\frac{{k}_{B}T}{h}e\left(\frac{\varDelta\:{G}^{o}}{RT}\right)$$

where *k*_*B*_ and *h* are Boltzmann and Planck’s constants, respectively. Accordingly, the enthalpy ($\:\varDelta\:{H}^{o}$ ) and the entropy ($\:\varDelta\:{S}^{o}$) of activation can be calculated using the following relationships:

$\:\varDelta\:{H}^{o}={E}_{a}-RT$ and $\:\varDelta\:{S}^{o}=(\varDelta\:{H}^{o}-\varDelta\:{G}^{o})/T$, respectively^58^. Table 3 summarized all the thermodynamic parameters derived from the Arrhenius and Eyring relations reveal the non-spontaneous nature of the oxidative oxidation process of PCT, as evidenced by the positive values of $\:\varDelta\:{G}^{o}$.

The data exhibited in Table 3 with the positive values Gibbs free energy of activation suggests that the reaction is endothermic, requiring energy input to proceed, consistent with photo-Fenton-like driven by photon energy. Notably, the degree of non-spontaneity increased with rising temperature. The positive enthalpy values (*ΔH°*) further confirmed the endothermic nature of the reaction. Moreover, the negative and relatively low entropy values (*ΔS*^*∘*^) suggest a decrease in the randomness of the system, likely due to restricted molecular motion or a more ordered transition state of the PCT species, while still maintaining a high yield of hydroxyl radicals^59^.

**Table 3:** Thermodynamic data for the removal of PCT using RTG-MIL-100(Fe)based photo-Fenton system.

Temp. K	E_a_ = 6.27 kJ/mol		
	$\:\varDelta\:{H}^{o}$	$\:\varDelta\:{S}^{o}$	$\:\varDelta\:{G}^{o}$
301	3.79	-258.07	80.94
313	3.67	-261.94	85.65
323	3.59	-263.41	88.66
333	3.51	-262.96	91.07

### Comprehensive evaluation of oxidation pathways, reactive species, and catalyst durability

#### Radical scavenging performance

**Fig. 9:**
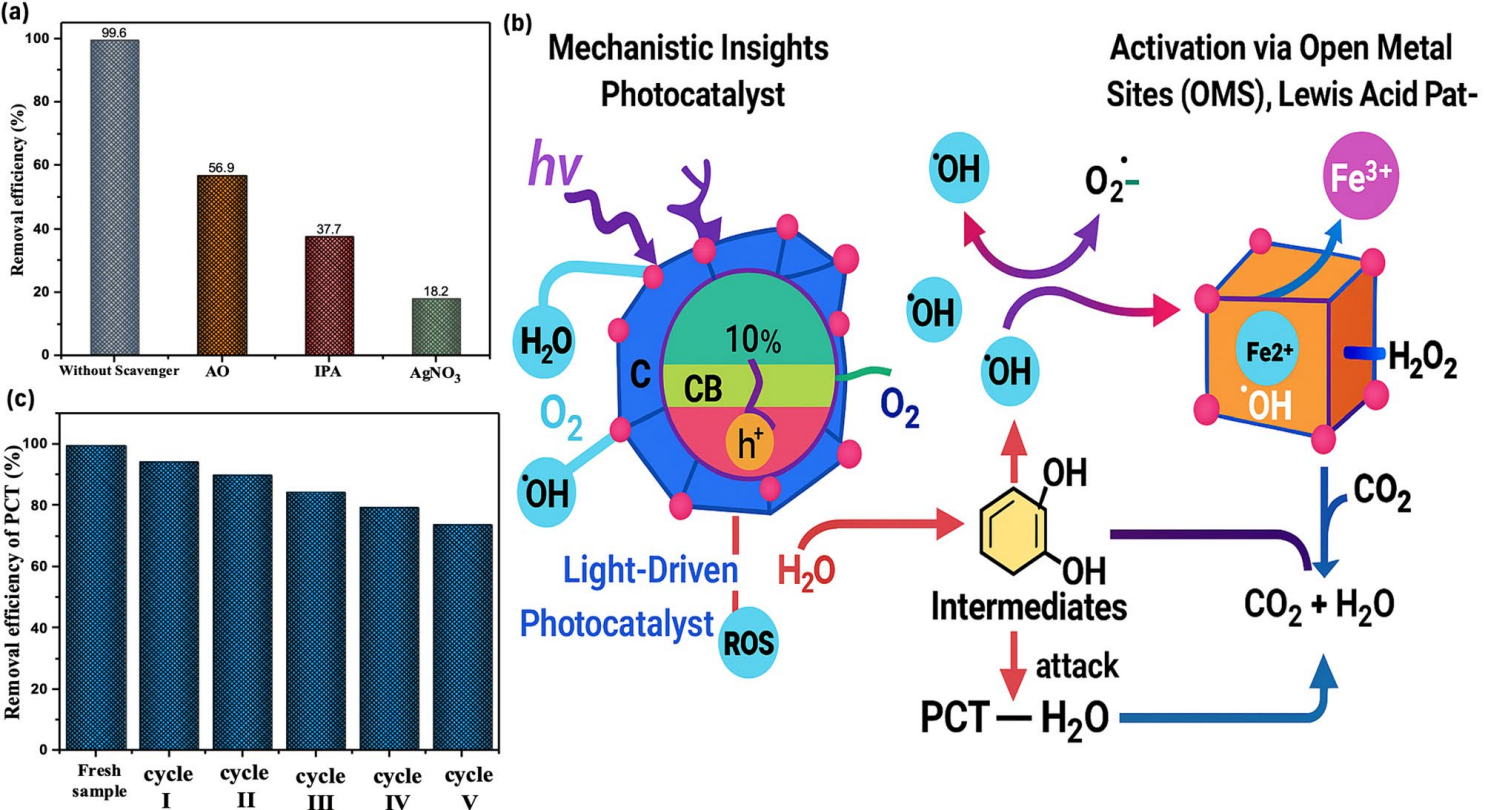
Reaction pathways of PCT oxidation over RTG-MIL-100(Fe)/H₂O₂/UV system: (**a**) Effect of radical scavengers on PCT oxidation, (**b**) Proposed dual-route oxidation mechanism of PCT oxidation over RTG-MIL-100(Fe)/H₂O₂/UV and (**c**) Reusability of RTG-MIL-100(Fe) over successive cycles.

To elucidate the dominant reactive species involved in the photocatalytic oxidation of PCT, radical scavenging experiments were conducted on the RTG-MIL-100(Fe)sample, which was selected due to its superior activity at the original pH and its facile room-temperature synthesis route. Various radical scavengers were employed, namely silver nitrate (AgNO_3_), isopropanol (IPA) and ammonium oxalate (AO) as shown in Fig. 9a. The oxidation efficiency in the absence of any scavenger reached 99.6%, indicating the high intrinsic activity of the catalyst under the optimized conditions. Upon the addition of AgNO₃, as known electron (e⁻) scavenger, the oxidation dropped significantly to 18.2%, highlighting the critical role of photogenerated electrons in initiating the oxidation process, either through direct reduction of O₂ or through the activation of H₂O₂ to form reactive oxygen species (ROS)^60^. In the presence of IPA (isopropanol), a well-established scavenger of hydroxyl radicals(^•^OH), the oxidation efficiency decreased to 37.3%, suggesting that ^•^OH plays a vital role in the oxidative oxidation of PCT. These radicals are primarily formed by the reaction of Fe²⁺ with H₂O₂ in a Fenton-like pathway, where the availability of Fe²⁺ ions is essential^61^. Notably, the enhanced Fe²⁺ content in the catalyst may be attributed to the role of KI, used during synthesis as a reducing agent, which promotes the reduction of Fe³⁺ to Fe²⁺^10^. Moreover, ammonium oxalate (AO), a hole (h⁺) scavenger, resulted in a moderate decrease in activity (to 56.9%), implying that photogenerated holes also participate in the oxidation, either through direct oxidation of paracetamol or through further generation of ROS^62^. Overall, the pronounced sensitivity of the system to both AgNO₃ and IPA underscores the dual-pathway mechanism of oxidation involving both electron- and •OH-mediated routes. This observation also reflects the indirect contribution of KI in improving catalytic performance by facilitating the Fe³⁺/Fe²⁺ conversion and thus accelerating Fenton-like reactions^63^.

#### Oxidation mechanism

Mechanistic Insights into PCT oxidation over RTG-MIL-100(Fe)/H_2_O_2_/UV system are assessed. The photocatalytic oxidation of PCT using RTG-MIL-100(Fe) is governed by a dual mechanism involving both photo-induced charge carriers and open metal sites (OMS). Light-Driven Photocatalysis (Semiconductor Behavior) has also occurred upon UV irradiation, MIL-100(Fe) behaves as a semiconductor and undergoes photoexcitation, where electrons (e⁻) are promoted from the valence band (VB) to the conduction band (CB), generating corresponding holes (h⁺) in the VB according to (Eq. 9).

9 $$\:\mathrm{R}\mathrm{T}\mathrm{G}-\mathrm{M}\mathrm{I}\mathrm{L}-100\left(\mathrm{F}\mathrm{e}\right)+\mathrm{h}\mathrm{v}\to\:\mathrm{R}\mathrm{T}\mathrm{G}-\mathrm{M}\mathrm{I}\mathrm{L}-100\left(\mathrm{F}\mathrm{e}\right)\:({\mathrm{e}}^{-}+{\mathrm{h}}^{+})$$

In such reaction, the photogenerated electrons (e⁻) can reduce H₂O₂ to form hydroxyl radicals (•OH), which are powerful oxidants. The holes (h⁺) can oxidize water or hydroxide ions to also generate •OH radicals^64^. This light-induced process plays a crucial role in generating reactive oxygen species (ROS) that attack and degrade PCT molecules. Suggested mechanism for oxidation of PCT by photocatalysis is displayed in the following equations:

10 $$\:{{h}^{+}+\:\:}^{.}OH\to\:{\:\:}^{.}OH$$

11 $$\:\:\:\:{{h}^{+}+OH}^{-}\to\:{\:\:}^{.}OH$$

12 $$\:{h}^{+}+\:{H}_{2}O\:\to\:{\:}^{.}OH+{H}^{+}$$

13 $$\:{{e}^{-}+O}_{2\:}\:\to\:\:{O}_{2}^{.-}$$

14 $$\:{{O}_{2}^{.-}+H}^{+}\to\:\:\:{\:}^{.}O{H}_{2}$$

15 $$\:{{O}_{2}^{.-}+H}^{+}\to\:{\:}^{.}O{H}_{2}$$

16 $$\:{\:}^{.}O{H}_{2}+{H}_{2}\mathrm{O}\to\:{H}_{2}{O}_{2}$$

17 $$\:{\:}^{.}OH+PCT\to\:\mathrm{i}\mathrm{n}\mathrm{t}\mathrm{e}\mathrm{r}\mathrm{m}\mathrm{e}\mathrm{d}\mathrm{i}\mathrm{a}\mathrm{t}\mathrm{e}\mathrm{s}\to\:{CO}_{2}\:+\:{H}_{2}\mathrm{O}$$

Further, the reaction is activated through open metal sites (OMS) via a Lewis acid pathway. Simultaneously, the Fe³⁺ centers in the RTG-MIL-100(Fe) framework, particularly those exposed at open metal sites during activation, act as Lewis acid sites that interact with H₂O₂ which behaves as an electron-donating Lewis base, leading to its activation as displayed in the following equations^65^:

18 $$\:{Fe}^{2+}\left(OMS\right)+{H}_{2}{O}_{2}\to\:{Fe}^{3+}\left(OMS\right)+{\:}^{.}OH\:{+\:OH}^{-}$$

19 $$\:{Fe}^{3+}\left(OMS\right)+{H}_{2}{O}_{2}\to\:{Fe}^{2+}+{\:}^{.}O{H}_{2}+{\mathrm{H}}^{+}$$

Overall, such introduced photo-Fenton-like reaction based on MOF farmwork redox reaction facilitates the continuous generation of •OH radicals. Thus, such trend is enhancing the oxidation of PCT even in the absence of light. Therefore, the synergistic action between UV-driven photogenerated charge carriers and Fe^2^⁺/Fe³⁺ OMS as Lewis acid sites enhances the efficiency of H₂O₂ decomposition and increases the total oxidative potential of the system. Schematic graphical illustration of the oxidation mechanism is displayed in Fig. 9b.

#### Catalyst reusability and metal leaching analysis stability

The reusability and stability of the RTG-MIL-100(Fe)catalyst are critical factors for its practical application in long-term wastewater treatment. To evaluate its catalytic durability, the used catalyst was recovered after each cycle by simple separation, followed by washing with distilled water and drying at 100 °C^66^. As depicted in Fig. 9c, the catalyst retained notable catalytic performance over 5 successive cycles, exhibiting only a 19% decrease in removal efficiency relative to the fresh catalyst. This demonstrates its structural integrity and sustained activity, highlighting the material’s potential for repeated use. Such recyclability affirms the catalyst’s applicability in real-world industrial wastewater treatment processes, supporting its promise as a sustainable and efficient photocatalyst.

Further, to evaluate the chemical stability of the RTG-MIL-100(Fe) catalyst under photo-Fenton-like operating conditions, Fe leaching was quantified using ICP–OES. For accurate detection, the leaching test was performed using a 1 L reaction volume containing 0.2 g of catalyst and 400 µM H₂O₂. The filtrate exhibited an Fe concentration of 2.6 mg/L, corresponding to approximately 2.6 mg of dissolved iron (about 1.3 wt% of the catalyst). This low leaching level indicates that the RTG-MIL-100(Fe) framework remains structurally and chemically stable during the reaction, confirming its suitability for practical and repeated applications. The corresponding Fe-leaching values demonstrating that only 1.3 wt% of Fe dissolved into the solution indicating excellent chemical stability of the RTG-MIL-100(Fe) framework. Hence, the dissolved iron in the treated water after the photo-Fenton reaction is 2.6 mg/L. Although this value is higher than the WHO aesthetic limit for drinking water (0.3 mg/L)^67^, it remains far below any toxicity-related thresholds and lies comfortably within the typical industrial discharge standards. Moreover, the measured Fe-leaching is markedly lower than the values commonly reported for MIL-100(Fe) and other Fenton/photo-Fenton catalysts, where iron dissolution typically ranges between 3 and 30 mg/L. These results collectively demonstrate that RTG-MIL-100(Fe) possesses excellent chemical stability with minimal iron loss during operation, confirming its robustness and suitability for practical photo-Fenton applications^68,69^.

### Comparative data with literature

To demonstrate the significance of the current study, it is essential to Compare it with the available exist data. In this regard, comparing different pharmaceutical pollutants treatment using Fenton based MOF systems is investigated and the data is tabulated in Table 4. According to the data displayed in the table, in comparison to other MIL-100(Fe)-based photocatalytic systems reported in Table 4, the KI-modified MIL-100(Fe) (RTG-MIL-100(Fe)) exhibits markedly superior performance, achieving the highest degradation efficiency (99.6%) among all these systems. Moreover, this near-complete removal is attained at the pollutant’s original pH (5.5) without any pH adjustment, whereas many previously reported MIL-100(Fe)-based systems required acidic conditions to achieve comparable performance. Additionally, the present system operates at an ultra-low catalyst dosage (20 mg/L), which is the lowest among those achieving over 90% removal, highlighting its exceptional catalytic efficiency. Another practical advantage is the simplicity of activation: in contrast to studies that employed high-intensity xenon arc lamps or other complex light sources, the RTG-MIL-100(Fe)delivers excellent performance under a standard UV lamp alone. Collectively, these features underscore the superior design and photocatalytic efficacy of the RTG-MIL-100(Fe)system compared to all previously reported MIL-100(Fe)-based counterparts.

**Table 4:** Photocatalytic performances of MIL-100(Fe) and RTG-MIL-100(Fe)for pharmaceutical pollutant removal.

Photocatalyst (MOF)	Activation method	Pollutant (Drug)*	Initial conc. (mg/L)	Catalyst dosage (mg/L)	Reaction time (min)	Removal (%)	Ref.
MIL-100(Fe)	Visible light	TC	20	100	120	72	^70^
MIL-100(Fe)	Photo-Fenton/UV	DCF	10	50	180	90	^71^
HF-MIL-100(Fe)	UV Lamp	TC	30	200	60	93.2	^72^
Quasi-MIL-100(Fe)	Photo-Fenton/UV	CIP	20	670	90	95.6	^73^
Fe_3_O_4_/MOS_2_/MIL-100(Fe)	Photo-Fenton/UV	OTC-HCl	-	-	100	90.92	^74^
MIL-100(Fe)/Ir-doped	Photo-Fenton/Xe lamp	TC	50	50	60	91	^75^
RTG-MIL-100(Fe)	Photo-Fenton/UV	PCT	10	20	120	99.6	This work

*TC: tetracycline; DCF: diclofenac; CIP: ciprofloxacin; OTC-HCl: oxytetracycline hydrochloride; PCT: paracetamol.

## Conclusion

This current work suggested a novel RTG-MIL-100(Fe) catalyst that was successfully synthesized through a facile, solvent-free, room-temperature with KI-assisted method and applied for the UV photo-Fenton oxidation of PCT. The direct incorporation of KI significantly promoted the Fe³⁺/Fe²⁺ redox cycle, thereby enhancing •OH radical generation and improving the overall photocatalytic performance. Process optimization revealed that nearly complete pollutant removal (99.6%) could be achieved under optimal conditions (20 mg/L and 400 ppm of catalyst and H₂O₂, respectively, using natural wastewater pH (5.5.) within 120 min of irradiance time. Furthermore, kinetic modeling confirmed that the oxidation followed a pseudo-first-order model. Comparative experiments highlighted the superior activity of the directly synthesized RTG-MIL-100(Fe) over post-synthetically modified counterparts, emphasizing the advantage of the direct synthesis route. The catalyst also demonstrated excellent reusability and structural stability across successive cycles, with ICP-OES confirming minimal iron leaching (2.6 mg/L). Overall, this work underscores the potential of KI-modified MIL-100(Fe) as an efficient and durable photo-Fenton-like catalyst for the treatment of pharmaceutical pollutants. The developed RTG-MIL-100(Fe) catalyst shows strong potential for integration into continuous-flow treatment units, decentralized wastewater treatment systems in hospitals, and advanced oxidation stages in pharmaceutical manufacturing plants. Its high reactivity under mild conditions suggests feasibility for scale-up in industrial wastewater remediation.

## Data Availability

The data supports the findings of this study are available from the corresponding author upon reasonable request.
